# A glycoside-specific glycosyltransferase transfers apiose to flavonoid glucosides in celery

**DOI:** 10.1093/plphys/kiad443

**Published:** 2023-08-05

**Authors:** Henryk Straube

**Affiliations:** Assistant Features Editor, Plant Physiology, American Society of Plant Biologists; Faculty of Science, Department of Plant and Environmental Sciences, Section for Plant Biochemistry, University of Copenhagen, 1871 Frederiksberg C, Copenhagen, Denmark

In 1843 a researcher reported the identification of an unusual glycosidic molecule that he named “apiin,” which he had isolated from celery (*Apium graveolens*; Braconnot 1843). Around 60 years later, another scientist noticed that mild hydrolysis of apiin yields an unusually branched pentose sugar, which he called “apiose” (Vongerichten 1901). All higher plants are assumed to contain apiose as an integral part of cell wall polysaccharides (Pičmanová and Møller 2016). Additionally, it is estimated that more than 100 plant species synthesize at least 1200 apiose-containing specialized metabolites, like flavonoids and cyanogenic glycosides (Pičmanová and Møller 2016).

Certain plant species, particularly in the Apiaceae family that includes celery and parsley (*Petroselinum crispum*), produce high amounts of apiose-containing flavonoids. Among the flavonoid glycosides that contain an apiose moiety, apigenin-7-*O*-β-D-apioglucoside (apiin) (Fig. 1A) is the most abundant. Luteolin-7-*O*-β-D-apioglucoside and chrysoeriol-7-*O*-β-D-apioglucoside are common in celery as well (Lin et al. 2007). Researchers also sporadically observed these compounds in other species from the families of Asteraceae, Fabaceae, Plantaginaceae, and Solanaceae (Watson and Orenstein 1975; Kashiwagi et al. 2005).

**Figure 1.**
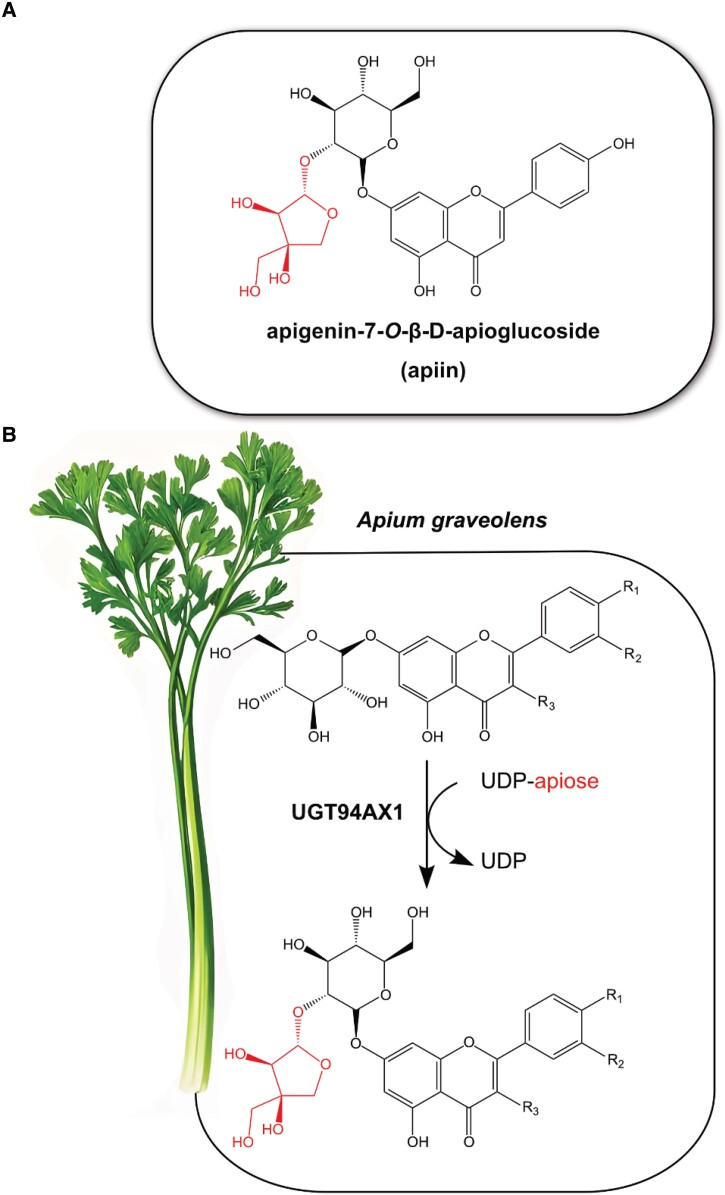
Apiosylation of different flavonoid 7-O-glucosides by celery UGT94AX1. **A)** The structure of apiin. **B)** UGT94AX1 transfers apiose from UDP-apiose to apigenin 7-*O*-glucoside (R_1_ = OH, R_2_, R_3_ = H) but also to other flavone and flavonol 7-*O*-glucosides, like quercetin 7-*O*-glucoside (R_1_, R_2_, R_3_ = OH), luteolin 7-*O*-glucoside (R_1_, R_2_ = OH, R_3_ = H), or chrysoeriol 7-*O*-glucoside (R_1_ = OH, R_2_ = OCH_3_, R_3_ = H). The apiose moiety is colored in red.

Although crude apiin was first described in 1843 (Braconnot 1843), its biosynthesis remained unclear. This is likely a result of the chemical instability of UDP-apiose, the apiose donor molecule in the apiosyltransferase reaction (Fujimori et al. 2019). Previously, a flavone 7-*O*-glucoside apiosyltransferase activity leading to the synthesis of apiin has been described in enzyme preparations from celery and parsley, but the underlying enzyme remained unknown (Ortmann et al. 1970, 1972).

In this issue of *Plant Physiology*, Yamashita et al. (2023) identified and characterized the celery apiosyltransferase UGT94AX1 that is highly specific in its transfer of apiose from uridine 5′-diphosphate-apiose to various flavonoid glucoside substrates (Fig. 1B).

To narrow down potential candidate genes for the sought-after apiosyltransferase, the researchers rationalized as follows. The apiose is attached to the glucose moiety of flavone 7-*O*-glucosides by a β1-2 linkage, so the enzyme transferring the sugar is likely a glycoside-specific glycosyltransferase (GGTs). The celery genome contains 26 potential GGTs, so the authors narrowed down the candidate genes by comparing the tissue-specific expression of these GGTs in celery RNAseq data with the content of apiin in the respective tissue. Yamashita and colleagues identified Agr35256, also named UGT94AX1, as a strong candidate gene and observed that the enzyme clusters in a phylogenetic tree with other GGTs that catalyze the formation of a β1-2 linkage. Strengthening these observations, the molecular mass and isoelectric point of the enzyme matched the previously partially purified apiosyltransferase from parsley (Ortmann et al. 1972).

As the researchers were initially searching for an apigenin-7-*O*-glucosides apiosyltransferase, they tested UGT94AX1 for this activity. Excitingly, the enzyme not only transferred an apiose from UDP-apiose to apigenin 7-*O*-glucoside but also to other flavone and flavonol 7-*O*-glucosides, like quercetin 7-*O*-glucoside, luteolin 7-*O*-glucoside, and chrysoeriol 7-*O*-glucoside. The enzyme had no detectable activity with other sugar-donor substrates. Interestingly, UGT94AX1 has a high affinity but a low turnover number for UDP-apiose. The authors speculate that this could be a trade-off during the neofunctionalization of the enzyme for UDP-apiose specificity. Intriguingly, the investigators identified 3 key amino acid residues for the enzyme's UDP-apiose specificity by comparing sequences of UGT94AX1 and other GGTs. They confirmed the residue involvement by site-directed mutagenesis and subsequent enzymatic activity comparisons of respective mutants with the wild-type enzyme, thus providing a structural basis for the first reported apiosyltransferase involved in plant specialized metabolism.

In summary, Yamashita et al. (2023) characterized UGT94AX1, an enzyme that is likely the sole apiosyltransferase in celery and showed that the enzyme transfers an apiose residue from UDP-apiose to various flavone 7-*O*-glucosides. UGT94AX1 thus likely catalyzes the last step of the apiin biosynthesis. As UGT94AX1 is potentially the only flavone 7-*O*-glucoside apiosyltransferase in celery, it will be exciting to see whether ablation of the respective gene leads to plants that lost their ability to synthesize apiosylated flavone 7-*O*-glucosides.

Little is known about the relevance of apiosylation, so the in vivo characterization of the relationship between the structure and function of the apiosylation and the analysis of involvement in eco-physiological relations will be most exciting (Kashiwagi et al. 2005; Pičmanová and Møller 2016; Yamashita et al. 2023). Additionally, the presented findings will inform approaches in synthetic biology and biotechnology to produce apiosylated flavone 7-*O*-glucosides.

## Dive Curated Terms

The following phenotypic, genotypic, and functional terms are of significance to the work described in this paper:
